# Role of seed priming using natural biostimulants in reducing salt stress effects by reshaping physio-biochemical and antioxidant defense systems in *glycine max* seedlings

**DOI:** 10.3389/fpls.2025.1630537

**Published:** 2025-10-13

**Authors:** Mostafa M. Rady, Amr Elkelish, Nada M. Nady, Sebnem Kusvuran, Alpaslan Kusvuran, Ahmed Shaaban, Haifa A S Alhaithloul, Mohamed A. M. Ali, Abdelghafar M. Abu-Elsaoud, Tapsoba François, Ali A. S. Sayed

**Affiliations:** ^1^ Botany Department, Faculty of Agriculture, Fayoum University, Fayoum, Egypt; ^2^ Biology Department, College of Science, Imam Mohammad ibn Saud Islamic University (IMSIU), Riyadh, Saudi Arabia; ^3^ Food and Agriculture Vocational School, Cankiri Karatekin University, Çankırı, Türkiye; ^4^ Agronomy Department, Faculty of Agriculture, Fayoum University, Fayoum, Egypt; ^5^ Biology Department, College of Science, Jouf University, Sakaka, Aljouf, Saudi Arabia; ^6^ Laboratory of Applied Biochemistry and Immunology, Department of Biochemistry and Microbiology, Joseph KI-ZERBO University, Ouagadougou, Burkina Faso

**Keywords:** glycine max, antioxidants, agronomic traits, osmoregulation, salinity, oxidative stress

## Abstract

Abiotic stress significantly damages crops, reducing global yields by over 50%. Among these challenges, salt stress poses a critical constraint that severely affects the growth, productivity, and quality of soybeans in various regions worldwide. Therefore, greenhouse pot experiments were conducted in the experimental farm of Fayoum University from May 1 to 15, 2024. Seed priming approach was performed using lemon fruit juice diluted to 4% (DLFJ_4%_) and bee honey diluted to 6% (DBH_6%_) as natural biostimulants. These biostimulants contain a wealth of growth-inducing compounds, including free amino acids, soluble sugars, antioxidants, vitamins, and essential nutrients. The purpose of this approach was to evaluate the effectiveness of DLFJ_4%_ or DBH_6%_ in mitigating the adverse effects of salt stress on the physio-biochemical and antioxidant defense systems in *Glycine max* seedlings. Salt stress was induced by irrigation with saline water, which was prepared by adding NaCl salt to normal water until EC = 8.60 dS m^–1^. The results showed that salt stress significantly increased superoxide (O_2_> ^•–^), hydrogen peroxide (H_2_O_2_), electrolyte leakage (EL), malondialdehyde, and ABA levels, which were linked to enhanced activity of antioxidant enzymes. Conversely, salt stress led to a substantial decrease in seed germination, seedling fresh and dry weights, and root activity. Furthermore, the photosynthetic and gas exchange parameters, leaf integrity traits, nutritional status, and hormonal levels of seedlings were all notably reduced. However, seed priming in DLFJ_4%_ or DBH_6%_ considerably alleviated the salinity-induced oxidative damage, leading to a notable decrease in O_2_^•–^, H_2_O_2_, malondialdehyde, EL, and ABA contents. These biostimulants further enhanced the activity of ROS-scavenging enzymes, including SOD, CAT, APX, and GR. This was accompanied by increased levels of soluble sugars, free proline, antioxidants, phytohormones, and essential macro- and micronutrients, as well as improved K^+^/Na^+^ and Ca^2+^/Na^+^ ratios. Both biostimulants nourished soybean seedlings and improved their morphological, physiological, and biochemical properties while also reducing salt stress toxicity. Overall, DBH_6%_ proved to be more effective than DLFJ_4%_. These findings suggest that DBH_6%_ is a potent natural biostimulant that enhances the salinity tolerance of salt-stressed soybean plants and improves sustainable agricultural practices.

## Introduction

Soybean (*Glycine max* L.) is the most economically important legume crop and one of the most nutritionally and commercially valuable lowland cash crops (Mishra et al., 2024). According to the FAO statistical database, soybeans are cultivated on approximately 133.8 million hectares (Mha), yielding a total seed production of 348.9 million tons (FAOSTAT, 2024). The seeds contain approximately 40% protein, 20% edible oil, and 30% carbohydrates, with 10% total sugar, making them essential for human, animal, fish, and poultry feeding (Siddiki et al., 2020; Mishra et al., 2024). Soybean-based products are rich in essential nutrients, making them an important dietary source of minerals. They provide a great way to obtain daily intake of minerals, including potassium (K^+^), sodium (Na^+^), magnesium (Mg), sulfur (S), and calcium (Ca^2+^) (Thabet Samar et al., 2021). It is used worldwide as an essential ingredient in bread, yogurt, soy milk, protein products, and edible oils (Elkelish, 2024; Elkelish and Abu-Elsaoud, 2024). Nonetheless, various biotic and abiotic stressors often hinder crop productivity, causing it to fall short of global demand. One of the unfavorable abiotic elements that reduces crop production and efficiency is salinity (Elkelish et al., 2021; Jabborova et al., 2021).

Soil salinization is a significant issue for farms and the global food supply. It is worsening at an estimated rate of approximately 10% per year, affecting 3,600 Mha out of 5,200 Mha of farmland. This problem results in an annual loss of about $27.5 billion (Alghamdi et al., 2023; Jabborova et al., 2023; Joshi et al., 2023). Prolonged accumulation of salinity-induced phytotoxic ions and transient osmotic stress are the first indications of salinity’s detrimental impacts on plants (Ullah et al., 2022, 2023). Due to climate change and inefficient farming practices, salinity stress is expected to become more prevalent. It negatively affects plant growth and productivity by causing osmotic and/or ionic stress (Makhadmeh et al., 2022b). During salinity stress, the excessive accumulation of sodium (Na^+^) and chloride (Cl^−^) ions leads to restricted nutrient uptake and ionic toxicity. Additionally, the reactive oxygen species (ROS) are overproduced within plant cells (Alsamadany et al., 2022; Hamdy et al., 2022). These negatively affect plant morphology, including poor seed germination, chlorosis (yellowing of leaves), and stunted growth. Various physio-biochemical changes, including oxidative stress, membrane disorganization, photoinhibition of photosystem II (PSII), and nutritional imbalances, are also induced (Balasubramaniam et al., 2023). Overproduction of ROS is the main mechanism through which salt induces oxidative damage; these ROS can harm DNA, lipids, and proteins (Makhadmeh et al., 2022a). In key cellular compartments such as the cytosol, endoplasmic reticulum, and mitochondria, various metabolic pathways can lead to the excessive production of ROS (Mandal et al., 2022; Abdou et al., 2023). The ROS include hydrogen peroxide (H_2_O_2_), superoxide anion (O_2_> ^•−^), hydroxyl radical (OH^−^), and singlet oxygen (^1^O_2_), which can disrupt different cellular processes (Kosar et al., 2022; Mohammed et al., 2023). ROS-induced oxidative stress damages cell membranes and decreases plant biomass accumulation. This oxidative stress interferes with several critical processes, including stomatal conductance, antioxidant defense mechanisms, osmotic adjustment, and the electron transport chain in both chloroplasts and mitochondria. Additionally, it leads to a reduction in chlorophyll and carotenoid contents and restricts the uptake of water and essential nutrients. Consequently, this leads to hindered photosynthetic machinery, particularly photosystem II (PSII) (Schierenbeck et al., 2023; Chauhan et al., 2023; Abd El-wahed et al., 2024).

To maintain salinity homeostasis and adjust osmotic balance, plants utilize various mechanisms, including the exclusion of toxic salt ions (Na^+^ and Cl^−^), the upregulation of both non-enzymatic and enzymatic antioxidants, and the accumulation of osmolytes such as proline, soluble sugars, glycine betaine, and amino acids (Thabet et al., 2024; Li et al., 2023). Non-enzymatic antioxidants include compounds such as phenolic compounds, flavonoids, free proline, ascorbate (AsA), glutathione (GSH), and α-tocopherol. Additionally, enzymatic antioxidants include superoxide dismutase (SOD), peroxidase (POD), ascorbate peroxidase (APX), glutathione reductase (GR), glutathione peroxidase (GPX), and polyphenol oxidase (PPO) (Tanveer and Ahmed, 2020; Ullah et al., 2023). Both osmolytes and antioxidants play crucial roles in regulating cell division, protecting plants from salinity-induced damage, preventing ion toxicity and chlorophyll loss, stabilizing cellular structures, and scavenging ROS (Singh et al., 2022; Ullah et al., 2022).

Given the detrimental effects of salinity on plant health and sustainable agriculture, which threaten global food security, there is an increasing interest in sustainable solutions such as natural biostimulants (NBSs). These NBSs are expected to become increasingly important in agriculture due to the rising global demand for sustainable agriculture practices (Kisvarga et al., 2022). They contain organic and inorganic constituents known to improve plant growth and development (Gupta et al., 2023; Ruzzi et al., 2024). They are an eco-friendly and safety-promising strategy for sustainable and clean farming under normal or stressful conditions (Belal et al., 2023). Diluted solutions of raw clover bee honey (DBH) and lemon juice (DLFJ) are novel NBSs that contain various growth-inducing biocomponents. These biocomponents promote plant growth and production under both stress-free and stressful conditions (Abou-Sreea et al., 2021; Alghamdi et al., 2022; Rady et al., 2023a).

The DLFJ contains a variety of organic acids, including citric, ascorbic, and malic acids, with a predominance of citric acid and ascorbate. Additionally, it includes a range of vitamins, minerals, sugars (including fructose, glucose, sucrose, and maltose), and nutritional components such as Ca^2+^, Mg, P, K^+^, and iodine (Chanukya et al., 2017; Rady et al., 2023a). Significant levels of flavonoids, flavones, and phenolic acids are also present in DLFJ (Klimek-Szczykutowicz et al., 2020). In a recent study, DLFJ significantly enhances growth characteristics, photosynthetic efficiency, gene expression, leaf integrity, and antioxidant activity (Rady et al., 2023a). Moreover, DLFJ mitigates the negative impacts of stress on plants by reducing the levels of H_2_O_2_, O_2_^•−^, electrolyte leakage (EL), and lipid peroxidation, commonly measured as malondialdehyde (MDA) content (Tahjib-Ul-Arif et al., 2021; Rady et al., 2023a).

Recent studies have shown that applying DBH to plants is one of the most effective NBSs, enhancing plant tolerance to unfavorable conditions (Semida and Rady, 2014; Semida et al., 2019). The key bioactive components of DBH, which include antioxidants, organic and inorganic acids, flavonoids, phenolic acids, vitamins, minerals, and osmoregulatory compounds (ORCs), enhance plants’ tolerance to stress. By applying DBH, all of its bioactive compounds can penetrate the plant tissue cells, enabling them to work effectively under both normal and stressful conditions (Alghamdi et al., 2022; Belal et al., 2023). The positive effects of this approach include enhanced water and nutrient uptake, increased levels of antioxidant compounds, reduced oxidative stress and damage, and alterations in the physio-biochemical mechanisms of the plants. Consequently, this leads to improved plant growth and productivity, as well as an accumulation of plant biomass (Abou-Sreea et al., 2021; Rady et al., 2023b).

There have been very few articles discussing the use of DLFJ and DBH as NBSs to enhance plant growth and development under stress. Additionally, DLFJ and DBH have not yet been utilized for cultivating *Glycine max* plants under conditions of salinity stress. This study tests the hypothesis that priming soybean seeds in either DLFJ or DBH solution at an appropriate concentration will improve the growth, development, and metabolic processes of salt-stressed soybean seedlings. This hypothesis is based on the findings of previous research (Abou-Sreea et al., 2021; Alghamdi et al., 2022; Rady et al., 2023a). This study aimed to investigate the potential benefits of soybean seed priming in either DLFJ or DBH solution at a concentration of 4% or 6%, respectively, on various aspects of salt-stressed *Glycine max* seedlings. Specifically, we focused on the following estimates: (I) seed germination, seedling growth, and nutritional status; (II) photosynthetic efficiency, leaf integrity, ROS such as O_2_> ^•−^ and H_2_O_2_, along with indices of stress damage, including MDA and EL; (III) gas exchange and phytohormone levels; and (IV) osmoregulatory compounds (ORCs), different antioxidant activities (both non-enzymatic and enzymatic), and overall total antioxidant activity.

## Materials and methods

### Growing conditions and sowing of plant material

Three pot experiments were undertaken in three adjacent open (net) greenhouses at the experimental farm of Fayoum University from May 1 to 15, 2024. One hundred eighty plastic pots, each with a diameter of 25 cm and a depth of 22 cm, were utilized for the three experiments, with 60 pots allocated to each experiment. Growing conditions included natural sunshine intensity, 26 ± 3/14 ± 2 °C mean day/night temperatures, 62 ± 4% humidity, and 12/12 h mean day/night photoperiods. The Egyptian Agricultural Research Center provided uniform, healthy soybean (*Glycine max* L.) seeds (cv. Giza-111). The sterilized seeds were rinsed for 0.5 min in a 70% (v/v) ethanol solution and for three min in a 0.25% NaClO solution. They were then rinsed several times with sterile deionized water (SD.H_2_O). Three seeds were sown in each pot in the three experiments. Before sowing, each pot was filled with 6 kg of growth medium (GM), as suggested by Rady and ur Rehman (2016) and Hassan et al. (2023), with a modification. The GM contained 16.5, 33.5, and 50.0% of acid-treated sand, vermiculite, and peat moss, respectively. Additionally, 0.25 g of humic acid was added to each pot. The sand was treated with 10% commercial HCl, rinsed three times over three days to remove both toxic and non-toxic ions. Afterwards, it was washed several times with SD.H_2_O. The GM was disinfected with Moncut SC (a fungicide, Central Glass Co., Ltd., Tokyo, Japan) at a rate of 0.125 g and then fertilized with 0.42 g NH_4_NO_3_ + 0.5 g CaH_4_P_2_O_8_ + 0.33 g K_2_SO_4_ + 0.83 g MgSO_4_ L^–1^. A 1.25 g CaCO_3_ L^−1^ and 2% acidified compost were added to adjust pH and increase GM fertility, respectively (Rady et al., 2023b).

### Treatments and trial setup

A total of 180 standard healthy seeds were allocated to each experiment, with 3 seeds sown in each pot. In each experiment, the 60 pots were divided into two equal sections, each containing 30 pots. The first section was specified for irrigation with normal water (NW; 1.60 dS m^–1^), while the second section was specified for irrigation with saline water (SW; 8.60 dS m^–1^). Each section was divided into three equal subsections, each containing 10 pots. In both sections, the first subsection was assigned for seed priming in SD.H_2_O, the second subsection was assigned for seed priming in a DLFJ**_4%_** solution [diluted lemon juice with 4.0% of the total soluble solids (TSS) in the juice; 8.82%), and the third subsection was assigned for seed priming in a DBH**_6%_** solution (diluted honey with 6.0% of the TSS in the honey; 83.8%). The NW was used to prepare the SW by adding NaCl salt up to EC = 8.60 dS m^–1^. After sterilizing the seeds, they were allowed to dry. Then, the seeds were primed for 8 h in SD.H_2_O, DLFJ**_4%_** solution, or DBH**_6%_** solution, as per the treatments. The seeds were dried overnight and sown in the early morning. Therefore, the treatments were as follows: (1) seed priming in SD.H_2_O + irrigation with NW (normal control; NCt), (2) seed priming in DLFJ**_4%_** + irrigation with NW, (3) seed priming in DBH**_6%_** + irrigation with NW, (4) seed priming in SD.H_2_O + irrigation with SW (saline control; SCt), (5) seed priming in DLFJ**_4%_** + irrigation with SW, and (6) seed priming in DBH**_6%_** + irrigation with SW. **Table 1** provides major chemical analysis of the lemon fruit juice (LFJ) and raw clover bee honey (BH).

**Table 1:** Chemical characteristics of the lemon fruit juice (LFJ), raw clover bee honey (BH), and their mixture (LFJ-BH; 5 mL of lemon juice + 5 g honey, respectively).

Components	Units	DLFJ	DBH
pH		3.10±0.20	4.38±0.24
Organic acids	%	5.92±0.31	0.51±0.03
Total soluble solids	°Brix	8.82±0.42	83.8±4.24
Total free amino acids	%	4.13±0.22	0.35±0.02
Total soluble sugars		1.18±0.05	81.3±4.02
Fructose		0.54±0.03	32.8±2.31
Glucose		0.49±0.02	39.4±2.77
Sucrose		0.03±0.00	2.48±0.20
Maltose		traces	4.55±0.26
Vitamin C		0.03±0.00	0.05±0.00
Total anthocyanins		0.01±0.00	0.05±0.00
Total phenols		0.12±0.01	0.13±0.00
Ash		0.34±0.01	0.39±0.02
Calcium	mg g^−1^ FW	0.08±0.00	0.09±0.00
Magnesium		0.07±0.00	0.09±0.00
Phosphorus		0.07±0.00	0.08±0.00
Potassium		0.07±0.03	0.08±0.00
Iodine		0.02±0.00	0.04±0.00
Antioxidant activity	mM Trolox eq. L^−1^	17.2±0.78	19.8±0.92

eq.; equivalent.

All trials were randomized and ended 15 days after seeding. Salt treatments-maintained GM salt concentration at EC = 8.60 dS m^–1^ and were monitored using ICP-AES (IRIS-Advan type, Thermo, USA). The pots were weighed and irrigated every 48 h. Each pot was irrigated to the soil’s capacity after weighing it to account for evapotranspiration. **Table 2** shows irrigation water chemistry. The pots were rotated and watered to mitigate local environmental changes for 15 days. Regular plant protection procedures were implemented to control weeds and diseases, following the recommendations of the Egyptian Agricultural Research Center. After 15 days, seedlings from the six treatments were collected for growth parameters, physio-biochemical characteristics, and antioxidant defense components. All samples were taken in the morning to avoid tissue damage from changing environmental conditions. The samples were quickly taken to laboratories for analysis.

**Table 2:** Chemical composition of irrigation water.

Concentration of ions (meq L^−1^)								EC (dS m^−1^)	Ph	SAR
CO_3_^2−^	HCO_3_^−^	SO_4_^2−^	Cl^−^	Mg^2+^	Ca^2+^	K^+^	Na^+^			
0.00	2.10	3.14	8.24	1.78	3.60	3.52	3.44	1.60	7.48	2.42

EC, electrical conductivity; and SAR, Sodium adsorption ratio.

### Preliminary experiments

To identify the best duration for priming soybean seeds and to identify the best concentrations of diluted lemon fruit juice (DLFJ) and diluted bee honey (DBH) for seed priming, three preliminary experiments were conducted from May 1 to May 15, 2023. **Supplementary Table S1** presents the ideal seed priming duration, which showed that an 8-h priming period at 25 ± 1 °C resulted in the highest seed germination percentage, as well as the greatest seedling fresh and dry weights and chlorophyll content. Consequently, an 8-hour priming duration was selected for the main study. Additionally, the most effective concentrations of DLFJ and DBH for seed priming, which yielded the best results for the parameters mentioned above, were found to be 4% DLFJ (DLFJ_4%_) and 6% DBH (DBH_6%_), compared to other concentrations of DLFJ and DBH (**Supplementary Tables S2–S3**). Therefore, DLFJ_4%_ and DBH_6%_ were used in the main study.

### Seed germination test

*Glycine max* seeds were disinfected using 70% (v/v) ethanol and 0.25% NaClO for 0.5 minutes and 3 min, respectively. The seeds were then washed multiple times with SD.H_2_O. A total of 4,800 healthy seeds and 120 Petri dishes, each with a diameter of 15 cm, were used. The dishes were divided into two main groups, with each group assigned to three different seed priming treatments. Forty seeds, primed in SD.H_2_O, DLFJ_4%_, or DBH_6%_, were placed on seven layers of Whatman No. 1 filter paper in each Petri dish. One group of 60 Petri dishes was assigned to irrigation with normal water (EC_w_ = 1.60 dS m^–1^), while the other group was assigned to irrigation with saline water (EC_w_ = 8.60 dS m^–1^). This resulted in six germination test treatments, each replicated 20 times (20 Petri dishes). This test was applied to both the preliminary and main studies. According to the treatment, each Petri dish received 20 mL of normal or saline water daily. All Petri dishes were incubated at 55 ± 3% humidity and 22 ± 1 °C. The experiment was designed using a completely randomized block design. The percentage of seed germination was recorded daily at a specific time. A seed was considered germinated when its radicle reached approximately 2 mm in length (Canavar et al., 2023).

### Assessment of growth and root activity

To determine the average fresh weight (g) per seeding, *G. max* seedlings were weighed 15 days after sowing. The seedlings were kept at 70 ± 2 °C until static dry weights reached the average (g) per seedling. To evaluate *G. max* root activity, the procedures of Ur Rehman et al. (2018) were followed. All seedling roots received 5 mL of 100 mM Na-P buffer (pH 7.0) in a 25-mL test tube, and the mixture was shaken for 30 min. In another test tube, the roots of another seedling were shaken for 3 h after adding 5 mL α-naphthylamine (α-NA). *ρ*-Aminobenzene sulfonic acid and NaNO_2_ (0.01%) were added at a rate of 1 mL of each to 1 μL of both solutions and then incubated at 30 °C for 10 min. Optical density was measured at 510 nm against the control without roots. In this method, the root contents of auto-oxidized α-NA were measured. α-NA contents were shaken for 30 min and 3 h and were evaluated by comparison with α-NA standard solutions. The oxidized α-NA was computed as a reduction in the α-NA amount from 30 min to 3 h. Root activity was calculated as the difference in oxidized α-NA content between the sample and control, expressed in μg g^–1^ fresh root h^–1^.

### Determination of photosynthetic parameters

Leaf contents of total chlorophyll (TChls) and total carotenoids (TCars) were measured in mg g^−1^ of fresh weight (FW). Fresh leaf samples weighing 1.0 g each were ground in 80% (v/v) acetone. The samples were then filtered and analyzed spectrophotometrically using a spectrophotometer (UV-160A, Shimadzu, Japan) at 662, 647, and 470 nm, and the contents of both TChls and TCars were calculated (Wellburn, 1994). The TChls content was calculated as the sum of chlorophyll ‘a’ and chlorophyll ‘b. Chlorophyll ‘a’ fluorescence (*F_v_/F_m_*) was assessed using a fluorometer (PAM-2000, Heinz-Walz) (Maxwell and Johnson, 2000). Fully grown leaves were used to assess chlorophyll fluorescence, including maximum primary photochemical quantum yield (*F_v_/F_0_*) and PSII photochemical efficiency (*F_v_/F_m_*). Additionally, PSII performance index (PI_ABS_) was assessed following the procedure of Clark et al. (2000).

### Determination of leaf integrity, and oxidant levels

The protocols of Rady and ur Rehman (2016); Osman and Rady (2014), and Rady (2011) were harnessed to measure leaf electrolyte leakage (EL, %), relative water content (RWC, %), and membrane stability (MSI, %), respectively.

To determine the EL (%), 20 leaf discs were prepared, and the electrical conductivity of 3 solutions (EC1, EC2, and EC3) was measured. EC1 was recorded immediately after the discs were prepared, EC2 was measured after heating the discs for 30 min at a temperature range of 45 °C to 55 °C, and EC3 was taken after boiling the discs for 10 min at 100 °C. The following formula was used:


$$EL\;(\%)=[\frac{\left(EC2-EC1\right)}{EC3}]\times 100$$

To determine the RWC (%), leaf-blade discs with a 2-cm diameter were prepared, and fresh (FW), turgid (TW), and dry weights (DW) were recorded. FW was taken immediately after preparing the discs, TW was taken after discs’ water saturation for 24 h, and DW was taken after drying for 48 h at 70 °C. Then, the following formula was used:


$$RWC\;(\%)=[\frac{\left(FW-DW\right)}{\left(TW-DW\right)}]\times 100$$

To determine the MSI (%), two 0.2 g leaf blade samples were prepared in two test tubes, each with 10 mL of distilled water. The EC of the two sample solutions (ECa and ECb) were measured. The ECa was taken after heating for 0.5 h at 40 °C, and the ECb was taken after boiling for 10 min at 100 °C. Then, the following formula was used:


$$MSI\;(\%)=[1-(\frac{ECa}{ECb})]\times 100$$

The levels of malondialdehyde (MDA), hydrogen peroxide (H_2_O_2_), and superoxide (O_2_^•−^) levels were evaluated by applying the Kubiś (2008); Velikova et al. (2000), and Madhava Rao and Sresty (2000) methods, respectively.

To estimate lipid peroxidation through MDA content, 100 mg of fresh leaf tissue was homogenized in 5 mL of a solution containing 0.07% NaH_2_PO_4_·2H_2_O and 1.6% Na_2_HPO_4_·12H_2_O (50 mM). The homogenate was then centrifuged at 20,000 × g for 25 min. The results for MDA were expressed as μmol g^−1^ FW.

To estimate H_2_O_2_ content, 0.25 g of fresh leaf tissue was homogenized in 5 mL of 5% trichloroacetic acid (TCA). Under cooling (4 °C), the homogenate was centrifuged at 12,000 ×g for 15 min. The supernatant was gathered, added to a reaction solution containing 10 mM potassium phosphate buffer (pH 7.0) and 1 M KI. The absorbance was read colorimetrically at 390 nm against H_2_O_2_ as a standard. The H_2_O_2_ content was expressed as μmol g^−1^ FW.

To estimate O_2_^•−^ content, 0.10 g of fresh leaf tissue was taken and cut into 1 × 1 mm fragments. At room temperature, the fragments were immersed in a solution containing 10 mM K-phosphate buffer, pH 7.8, 0.05% NBT, and 10 mM NaN_3_ for 1 h. Two mL of immersed solution was heated at 85 ^○^C for 15 min and cooled rapidly. Absorbance was measured calorimetrically at 580 nm. The O_2_^•−^ content was expressed as μmol g^−1^ FW.

### Determination of gas exchange parameters and nutrient contents

By using the fully expanded leaf tissues from the top of plants with an LCA-4 infrared gas analyzer (Anal. Dev. Co., Hoddesdon, England), measurements of the net rates of photosynthesis (*P_n_*), transpiration (*E*), and CO_2_ assimilation (*A*), as well as the conductance of stomata (*gs*) were performed meticulously. Contents of macro-nutrients (N, P, and K^+^) were determined in dried *G. max* leaf powder. The micro-Kjeldahl system was harnessed to evaluate the total N content. Colorimetrically, the stannous chloride-ammonium molybdate reagent was applied to assess P content after extraction by NaHCO_3_ (Olsen et al., 1954; King and Wotton, 1957). An ELE Flame Photometer system (Leighton Buzzard, UK) was applied to evaluate K^+^ and Na^+^ content. An Atomic Absorption Spectrophotometry system was also used to assess Ca^2+^, Fe^2+^, Mg^2+^, Mn^2+^, and Zn^2+^ contents (Chapman, 1965).

### Determination of osmoregulatory and antioxidant compound contents

Total soluble sugar content (mg g^−1^ DW) was determined (Irigoyen et al., 1992). After extraction and centrifugation, the supernatant was collected to mix with a freshly-prepared anthrone. The mixture was incubated for 10 min at 100 °C. Using a UV-160A UV–vis spectrometer (Shimadzu, Kyoto, Japan), the absorbance was measured at 625 nm, and the total soluble sugar content was calculated using a standard curve prepared with glucose.

The method of Bates et al. (1973) was followed to determine free proline content (μM g^–1^ DW). After extraction and centrifugation, the supernatant was mixed with a freshly prepared acid-ninhydrin solution. After incubation for 0.5 h at 90 °C, the reaction was terminated in an ice bath. The samples were extracted again with toluene to obtain the toluene phase. Using a UV-160A UV–vis spectrometer (Shimadzu, Kyoto, Japan), the absorbance of the toluene phase was measured at 520 nm, and the free proline content was calculated using a standard curve prepared with proline.

The methods of Kampfenkel et al. (1995) were used to determine the ascorbate (AsA) content (μM g^− 1^ FW) and the AsA redox state (%). A mixture was prepared containing K-P buffer (30 mM, pH 7.4), 2.5% TCA, 8.4% H_3_PO_4_, 0.8% bipyridyl, and 0.3% FeCl_3_. The leaf extract was added to this mixture and incubated at 40 °C for 30 min. The absorbance was then taken at 525 nm. The DHA (dehydro-AsA) + AsA levels were evaluated after reacting to the extract with 0.5 mM DTT to evaluate a total reduction of AsA by recording the absorbance at 525 nm versus a standard (L-AsA). The following formula was then applied:


$$\text{AsA redox state}\;(\%)=[\frac{\text{AsA}}{\text{AsA}+\text{DHA}}]\times 100$$

The methods of Griffith (1980) were used to assess the glutathione (GSH) content (μM g^− 1^ FW) and the GSH redox state (%). A mixture was prepared containing Na-P buffer (0.13 M, pH 7.4), Na-P buffer (7 mM, pH 6.8), and DTNB (6 mM). The leaf extract was added to this mixture and incubated at 30 °C for 10 min. The absorbance was then taken at 412 nm. After reducing the GSSG to GSH, the total GSH content was measured. The GSSG reduction was performed by adding the leaf extract to a sodium-phosphorus buffer (0.13 M, pH 7.4) + 1 unit of GSH reductase. The absorbance was read at 412 nm. The GSH and GSSG + GSH levels were evaluated versus a GSH standard. The following formula was then applied:


$$\text{GSH redox state}\;(\%)=[\frac{\text{GSH}}{\text{GSH }+\text{ GSSG}}]\times 100$$

### Assaying of enzyme and total antioxidant activity

The extraction protocol developed by Mukherjee and Choudhuri (1983) was utilized to extract a 0.5 g leaf sample for enzyme activity assays. After the homogenization process, the mixture was centrifuged at 15,000 × g for 10 min. The resulting supernatant was then collected and used for the assays. Activity of superoxide dismutase (SOD), glutathione reductase (GR), catalase (CAT), and ascorbate peroxidase (APX) was evaluated by applying the techniques of Giannopolitis and Ries (1977); Smith et al. (1988); Ali et al. (2019), and Asada (2006), respectively. For the SOD activity assay, each reaction mixture contained 400 μL of H_2_O_2_, 250 μL of buffer, 100 μL of methionine, 100 μL of Triton, 50 μL of NBT, 50 μL of enzyme extract, and 50 μL of riboflavin. The samples were kept under light (60 W) for 15 min. Enzyme activity was recorded at 560 nm using a UV-160A UV–vis spectrometer (Shimadzu, Kyoto, Japan). The method for assessing GR activity involves measuring the increase in absorbance at 412 nm resulting from the reduction of 5,5′-dithiobis(2-nitrobenzoic acid) by GSH in the sample supernatant. The activity of CAT was measured in a 3 ml reaction solution containing a P-buffer (pH 7.8), H_2_O_2_, and the enzymatic extract. A decrease in absorbance at 240 nm indicates enzyme activity. The activity of APX was evaluated in a reaction solution with a K-P-buffer (pH 7.0), H_2_O_2_, AsA, and the enzyme extract. The reduced value in the absorbance at 240 nm indicates APX activity. The activity of all enzymes was expressed as “units’ g^− 1^ protein”. The method of Bradford (1976) was harnessed to evaluate soluble protein content.

The antioxidant activity of *G*. *max* leaves was determined by the DPPH (2,2-diphenyl-1-picrylhydrazyl)-free radical scavenging activity test (Brand-Williams et al., 1995). A sample of 1.0 mL extract was appended to 1 mL of 20 mg L^−1^ DPPH solution, and CH_3_OH (methanol) was used as a blank. The mixture was incubated in the dark for 20 min. The absorbance was read Spectrophotometrically (UV–VIS) at 517 nm. Free radical scavenging activity (as DPPH free radical inhibition, %) was computed by using the following formula:


$$Inhibition\;(\%)=[\frac{Blank\;absorbance-Sample\;absorbance}{Blank\;absorbance}]\times 100$$

### Determination of phytohormone levels

The Perkin-Elmer GC–MS system (Waltham, MA, USA) was utilized to quantify indole-3-acetic (IAA), gibberellic acid (GA_3_), and cytokinins (CKs) (Nehela et al., 2016; Rady et al., 2019). Leaf samples, 0.2g each, were extracted in an ice-cold mixture (0.1% HCl: 19.9% H2O: 80% CH_3_OH, v/v/v). The extracts were centrifuged at 25,000 ×g for 5 min. The resulting supernatants were concentrated under a N stream to 50 μL and then stored at − 80 °C. For IAA, the supernatants were derivatized, and the organic phase was then dehydrated with Na_2_SO_4_. For both GA_3_ and CKs, the concentrated supernatants were derivatized and dehydrated. The IAA, GA_3_, and CKs were identified utilizing authentic standards. The abscisic acid (ABA) was extracted with 12:5:3 (v/v/v) CH_3_OH: CHCl_3_: 2N NH_4_OH, and the ABA content was estimated by an HPLC system (Ünyayar et al., 1996).

### Statistical analysis

The two-way analysis of variance (ANOVA) technique was executed after testing for homoscedasticity (Gomez and Gomez, 1984). Treatment means were compared using the least significant difference (LSD) test at *p* ≤ 0.05 with CoStat software (version 6.29; CoHort Software). Statistical computations were carried out using NCSS and Microsoft Excel^®^ (2013) (Snedecor and Cochran, 1989).

## Results

### Impacts of DLFJ_4%_ or DBH_6%_ on the seed germination and growth of soybean seedlings

**Figure 1:**
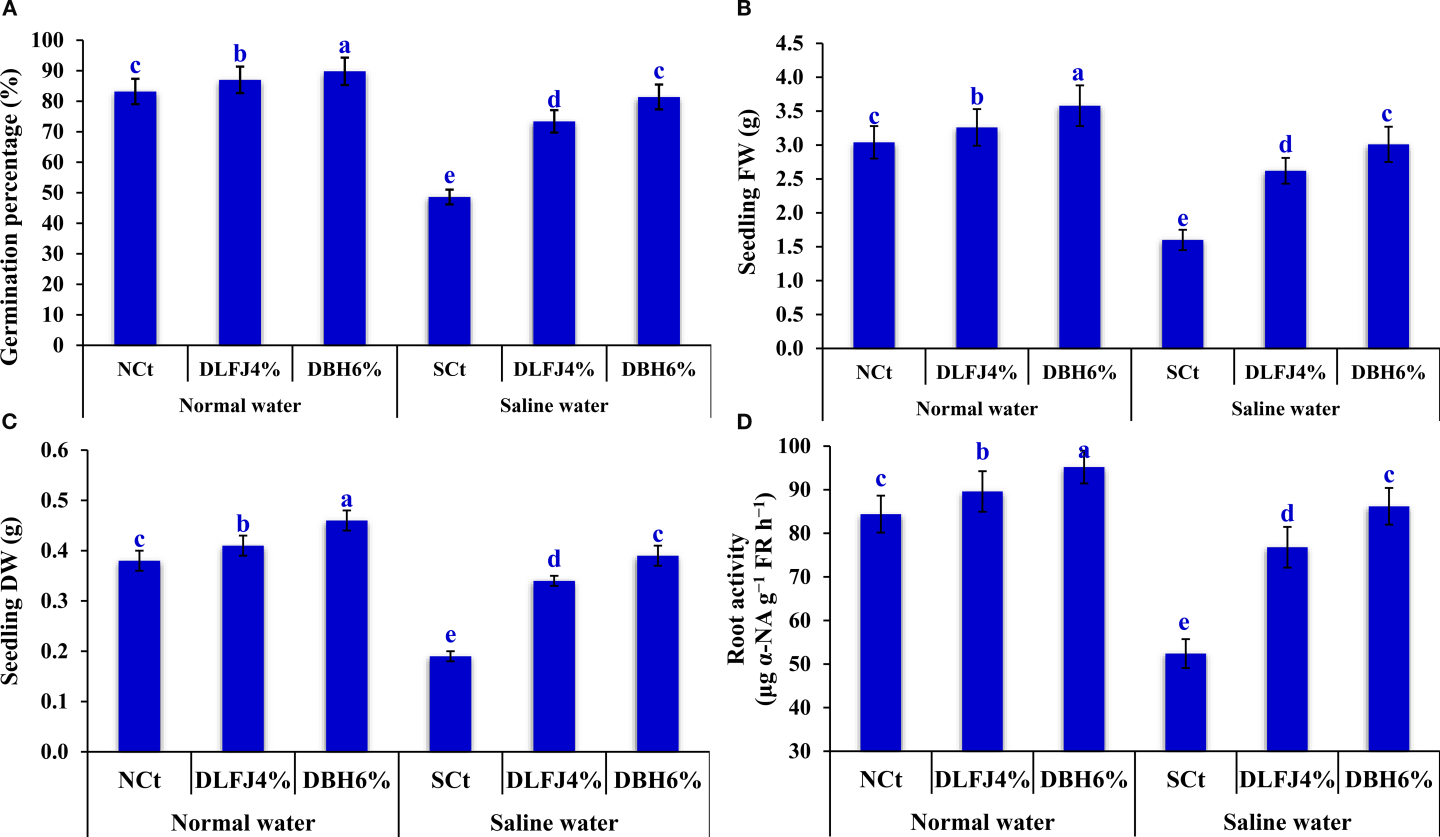
Response of germination percentage **(A)**, seedling FW **(B)**, seedling DW **(C)**, and root activity **(D)** of soybean seedlings (*Glycine max* L., cv. Giza-111) grown under irrigation with normal water (EC_w_= 1.60 dS m^–1^) or saline water (EC_w_= 8.60 dS m^–1^) to soaking the seeds in DLFJ_4%_ or DBH_6%_. Based on the two-way ANOVA conducted across all six treatment combinations (i.e., irrigation water saline × foliar biostimulant) and the LSD test, bars (mean ± standard error; n = 3 for each) labeled with similar letters in the same column did not differ significantly at a *p* ≤ 0.05 level of probability. NCt, normal control; where seeds were soaked and then irrigated using normal water, SCt, saline control; where seeds soaked in normal water and then irrigated with saline water, DLFJ_4.0%_, soaking the seeds in diluted lemon juice at a concentration of 4.0%; DBH_6.0%_, soaking the seeds in diluted bee honey solution at a concentration of 6.0%; GP, the germination percentage measured when the seed radicle reaches about 2 mm in length; FW, fresh weight; DW, dry weight; α-NA, α-naphthylamine; FR, fresh root; and EC_w_, irrigation water salinity.

Salinity stress significantly reduced the percentage of seed germination (GP, %), the fresh weight of seedlings (SFW, g), the dry weight of seedlings (SDW, g), and the root activity (RA, %) in soybean seedlings. However, priming soybean seeds in DLFJ_4%_ and DBH_6%_ improved these parameters and mitigated the salinity-induced deterioration (**Figure 1**). Compared to irrigation with normal water (normal control; NCt), soybean seedlings irrigated with saline water experienced a decline of 42% in GP, 47% in SFW, 50% in SDW, and 38% in RA. However, soybean seeds primed in DLFJ_4%_ and DBH_6%_, and irrigated with normal water, recorded an increase of 5% and 8% in GP, 7% and 18% in SFW, 8% and 21% in SDW, and 6% and 13% in RA, respectively, compared to the NCt (irrigation with normal water without seed priming in biostimulants). Interestingly, priming soybean seeds in DLFJ_4%_ and DBH_6%_, and irrigated with saline water, recorded an increase of 51% and 67% in GP, 64% and 88% in SFW, 79% and 105% in SDW, and 47% and 65% in RA, respectively, compared to the saline control (SCt; irrigation with saline water without seed priming in biostimulants).

### Impacts of DLFJ_4%_ or DBH_6%_ on photosynthetic parameters and leaf integrity

Irrigation of soybean seedlings with saline water significantly deteriorated photosynthesis, estimated as total chlorophyll (TChls), total carotenoids (TCars), the quantum efficiency of PSII (Fv/Fm), and the performance index (PI_ABS_). Additionally, leaf integrity, measured as relative water content (RWC) and membrane stability index (MSI), was noticeably disturbed. However, seed priming in DLFJ_4%_ and DBH_6%_ significantly enhanced all of these parameters and alleviated the negative effects of salinity (**Table 3**). Irrigating seedlings with saline water resulted in a decline of 57% in TChls, 50% in TCars, 42% in *F_v_/F_m_*, 46% in PI_ABS_, 42% in RWC, and 49% in MSI compared to the NCt. However, seed priming in DLFJ_4%_ and DBH_6%_ and irrigating with normal water led to an increase of 15% and 27% in TChls, 8% and 18% in TCars, 6% and 11% in *F_v_/F_m_*, 9% and 19% in PI_ABS_, 4% and 6% in RWC, and 5% and 9% in MSI, respectively, compared to the NCt. Furthermore, seed priming in DLFJ_4%_ and DBH_6%_ and irrigated with saline water, there was a significant increase of 61% and 124% in TChls, 41% and 91% in TCars, 48% and 67% in *F_v_/F_m_*, 52% and 80% in PI_ABS_, 42% and 70% in RWC, and 64% and 94% in MSI, respectively, compared to the SCt.

**Table 3:** Response of photosynthetic parameters of soybean (*Glycine max* L., cv. Giza-111) seedlings grown under irrigation with normal water (NW; EC_w_ = 1.60 dS m^–1^) or saline water (SW; EC_w_ = 8.60 dS m^–1^) to soak the seeds in diluted lemon fruit juice (DLFJ) or diluted bee honey (DBH) at 4.0 or 6.0%, respectively.

Treatments^1^		TChls	TCars	*F_v_/F_m_*	PI_ABS_	RWC	MSI
Irrw	Bio-ss	(mg g^–1^ FW)				(%)	
NW	NCt	2.13±0.09**^c^**	0.44±0.02**^c^**	0.79±0.03**^c^**	15.1±0.31**^c^**	86.2±3.84**^b^**	62.4±3.2**^c^**
	DLFJ**_4.0_**	2.44±0.11**^b^**	0.48±0.02**^b^**	0.84±0.04**^b^**	16.4±0.33**^b^**	89.9±4.20**^a^**	65.4±3.3**^b^**
	DBH**_6.0_**	2.70±0.12**^a^**	0.52±0.03**^a^**	0.88±0.04**^a^**	17.9±0.35**^a^**	91.1±4.45**^a^**	67.8±3.6**^a^**
SW	SCt	0.92±0.05**^e^**	0.22±0.01**^e^**	0.46±0.02**^e^**	8.22±0.18**^e^**	49.8±2.22**^d^**	31.6±1.8**^e^**
	DLFJ**_4.0_**	1.48±0.07**^d^**	0.31±0.02**^d^**	0.68±0.03**^d^**	12.5±0.24**^d^**	70.6±3.20**^c^**	51.8±2.3**^d^**
	DBH**_6.0_**	2.06±0.08**^c^**	0.42±0.03**^c^**	0.77±0.03**^c^**	14.8±0.30**^c^**	84.8±3.88**^b^**	61.2±3.1**^c^**
*p*-value		<0.001**	0.001**	<0.001**	0.050*	0.035*	0.025*

**^1^** Based on the LSD test, mean values (± standard errors) followed with similar letters in the same column not differed significantly at *p* ≤ 0.05 level of probability. IrrW; irrigation water, Bio-Ss; biostimulators, EC_w_; irrigation water salinity, NCt; normal control, where seeds were soaked and then irrigated using normal water, SCt; saline control, where seeds soaked in normal water and then irrigated with saline water, DLFJ_4.0%_; soaking the seeds in diluted lemon juice at a concentration of 4.0%, DBH_6.0%_; soaking the seeds in diluted bee honey solution at a concentration of 6.0%, TChls, total chlorophylls; TCars, total carotenoids; FW, fresh weight; *F_v_/F_m_*, chlorophyll “a” fluorescence; PI_ABS_, photosynthetic performance index; RWC, relative water content; and MSI, membrane stability index. *p < 0.05 (statistically significant); **p < 0.01 (highly statistically significant).

### Impacts of DLFJ_4%_ or DBH_6%_ on oxidant levels and their damage in soybean

**Figure 2:**
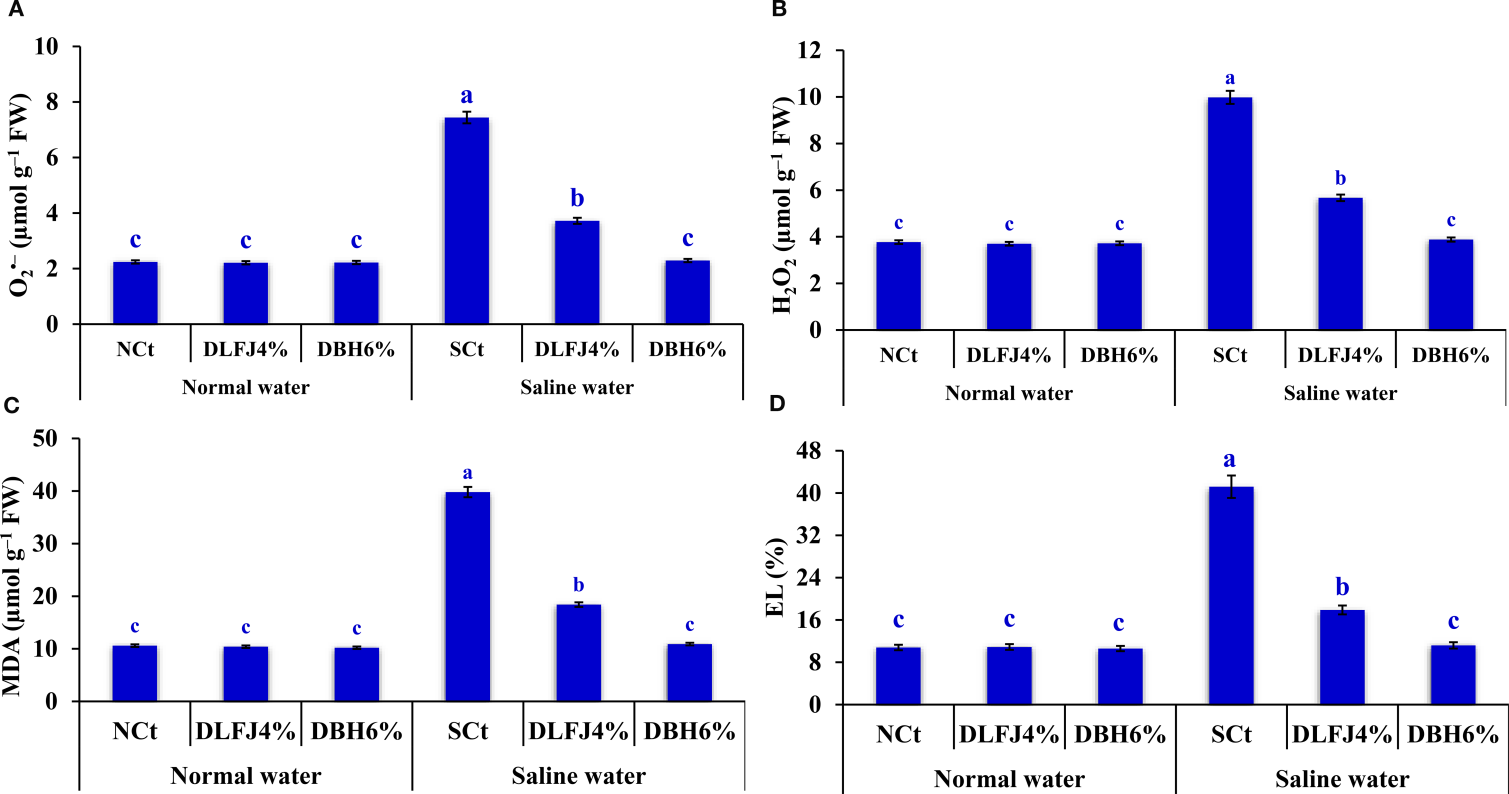
Response of oxidant levels and their damage; O_2_^•–^ **(A)**, H_2_O_2_ **(B)**, MDA **(C)**, and EL **(D)** in soybean seedlings (*Glycine max* L., cv. Giza-111) grown under irrigation with normal water (EC_w_= 1.60 dS m^–1^) or saline water (EC_w_= 8.60 dS m^–1^) to soaking the seeds in DLFJ_4%_ or DBH_6%_. Based on the two-way analysis of variance conducted across all six treatment combinations (i.e., irrigation water saline × foliar biostimulant) and the LSD test, bars (mean ± standard error; n = 3 for each) labeled with similar letters in the same column did not differ significantly at a *p* ≤ 0.05 level of probability. NCt, normal control; where seeds were soaked and then irrigated using normal water, SCt, saline control; where seeds soaked in normal water and then irrigated with saline water, DLFJ_4.0%_, soaking the seeds in diluted lemon juice at a concentration of 4.0%; DBH_6.0%_, soaking the seeds in diluted bee honey solution at a concentration of 6.0%; GP, the germination percentage measured when the seed radicle reaches about 2 mm in length; O_2_^•–^, superoxide; H_2_O_2_, hydrogen peroxide; MDA, malondialdehyde; EL, electrolyte leakage; FW, fresh weight; and EC_w_, irrigation water salinity.

Salinity stress led to an increase in oxidants, such as superoxide (O_2_> ^•–^) and hydrogen peroxide (H_2_O_2_) levels. This resulted in significant oxidant damage, which was evaluated by measuring lipid peroxidation through malondialdehyde (MDA) content and electrolyte leakage (EL) in soybean seedlings. However, seed priming in DLFJ_4%_ and DBH_6%_ markedly reduced the levels of oxidants and their associated damage, thereby alleviating the negative effects of salinity (**Figure 2**). Compared to irrigation with normal water (NCt), seedlings irrigated with saline water experienced an increase of 232% in O_2_> ^•–^, 165% in H_2_O_2_, 275% in MDA, and 281% in EL. However, priming soybean seeds in DLFJ_4%_ and DBH_6%_, and irrigated with normal water, led to a slight reduction of 1% and 1% in O_2_^•–^, 2% and 1% in H_2_O_2_, 2% and 4% in MDA, and 1% and 2% in EL, respectively, compared to the NCt. Moreover, seed priming in DLFJ_4%_ and DBH_6%_, and irrigated with saline water, resulted in a decrease of 50% and 49% in O_2_^•–^, 43% and 61% in H_2_O_2_, 54% and 73% in MDA, and 57% and 73% in EL, respectively, compared to the SCt.

### Impacts of DLFJ_4%_ or DBH_6%_ on gas exchange parameters of soybean

**Figure 3:**
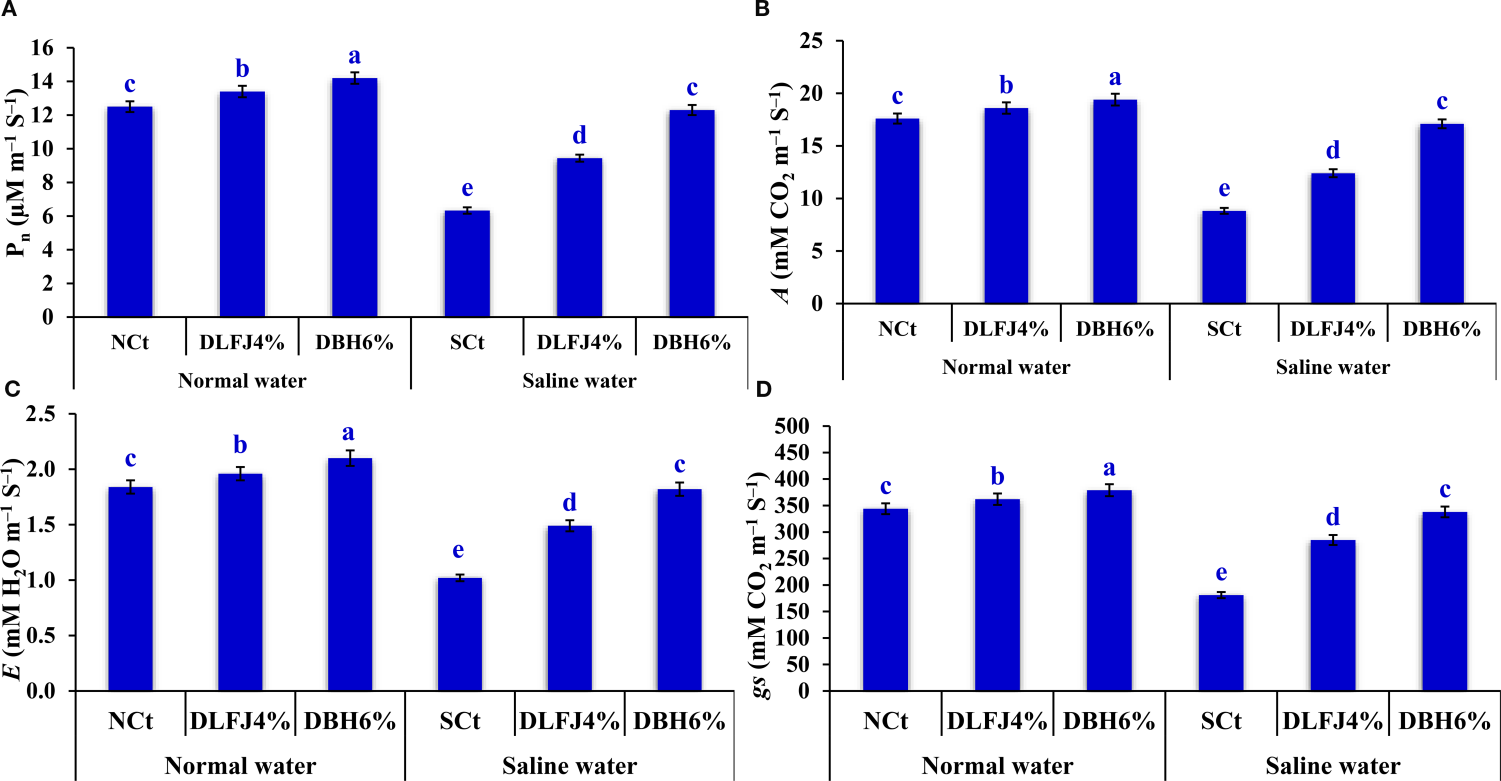
Response of gas exchange parameters; *P_n_* **(A)**, *A* **(B)**, *gs* **(C)**, and *E* **(D)** of soybean seedlings (*Glycine max* L., cv. Giza-111) grown under irrigation with normal water (EC_w_ = 1.60 dS m^–1^) or saline water (EC_w_ = 8.60 dS m^–1^) to soaking the seeds in DLFJ_4%_ or DBH_6%_. Based on the two-way analysis of variance conducted across all six treatment combinations (i.e., irrigation water saline × foliar biostimulant) and the LSD test, bars (mean ± standard error; n = 3 for each) labeled with similar letters in the same column did not differ significantly at a *p* ≤ 0.05 level of probability. NCt, normal control; where seeds were soaked and then irrigated using normal water, SCt, saline control; where seeds soaked in normal water and then irrigated with saline water, DLFJ_4.0%_, soaking the seeds in diluted lemon juice at a concentration of 4.0%; DBH_6.0%_, soaking the seeds in diluted bee honey solution at a concentration of 6.0%; GP, the germination percentage measured when the seed radicle reaches about 2 mm in length; *P_n_*, net photosynthesis rate; *A*, CO_2_ assimilation rate; *gs*, stomatal conductance; *E*, transpiration rate; and EC_w_, irrigation water salinity.

Irrigating soybean seedlings with saline water negatively impacted key gas exchange parameters, including net photosynthesis rate (*Pn*), CO_2_ assimilation rate (*A*), stomatal conductance (*gs*), and transpiration rate (*E*). However, seed priming in DLFJ_4%_ and DBH_6%_ significantly improved these gas exchange parameters, helping to alleviate the adverse effects of salinity (**Figure 3**). Irrigating seedlings with saline water resulted in a decrease of 49% in *Pn*, 50% in *A*, 47% in *gs*, and 45% in *E* compared to the NCt. However, seed priming in DLFJ_4%_ and DBH_6%_ and irrigating with normal water led to an increase of 7% and 14% in *Pn*, 6% and 10% in *A*, 5% and 10% in *gs*, and 7% and 14% in *E*, respectively, compared to the NCt. Furthermore, seed priming in DLFJ_4%_ and DBH_6%_ and irrigated with saline water led to a significant increase of 49% and 94% in *Pn*, 41% and 94% in *A*, 57% and 87% in *gs*, and 46% and 78% in *E*, respectively, compared to the SCt.

### Impacts of DLFJ_4%_ or DBH_6%_ on osmoregulatory and antioxidant contents of soybean

Salinity stress significantly increased the accumulation of osmoregulatory and antioxidant contents, including total soluble sugars (TSsg), free proline (FPro), ascorbate (AsA), and glutathione (GSH). This resulted in significant increases in the redox states of AsA and GSH in soybean seedlings. Seed priming in DLFJ_4%_ and DBH_6%_ further increased the contents of osmoregulatory and antioxidant compounds, as well as the redox states of AsA and GSH, contributing to the alleviation of the negative effects of salinity (**Table 4**). Compared to irrigation with normal water (NCt), seedlings irrigated with saline water led to an increase of 16% in TSsg, 32% in FPro, 28% in AsA, 22% in GSH, 14% in AsA redox state, and 25% in GSH redox state. Moreover, priming soybean seeds in DLFJ_4%_ and DBH_6%_, and irrigated with normal water, resulted in an increase of 4% and 19% in TSsg, 7% and 16% in FPro, 7% and 18% in AsA, 7% and 22% in GSH, 5% and 10% in AsA redox state, and 5% and 16% in GSH redox state, respectively, compared to the NCt. Furthermore, seed priming in DLFJ_4%_ and DBH_6%_, and irrigated with saline water, resulted in an increase of 7% and 24% in TSsg, 10% and 24% in FPro, 7% and 17% in AsA, 9% and 31% in GSH, 7% and 12% in AsA redox state, and 8% and 21% in GSH redox state, respectively, compared to the SCt.

**Table 4:** Response of osmoregulatory and antioxidant compound contents of soybean (*Glycine max* L., cv. Giza-111) seedlings grown under irrigation with normal water (NW; EC_w_ = 1.60 dS m^–1^) or saline water (SW; EC_w_ = 8.60 dS m^–1^) to soak the seeds in diluted lemon fruit juice (DLFJ) or diluted bee honey (DBH) at 4.0 or 6.0%, respectively.

Treatments^1^		TSsg content	FPro content	Asa content	GSH content	Asa redox state	GSH redox state
Irrw	Bio-ss	Mg g^–1^ DW	µm g^–1^ DW	µm g^–1^ FW		%	
NW	NCt	12.4±0.32**^f^**	33.6±0.89**^f^**	2.16±0.04**^f^**	1.02±0.03**^e^**	68.8±1.38**^f^**	21.0±0.44**^f^**
	DLFJ**_4.0_**	12.9±0.34**^e^**	35.8±0.93**^e^**	2.32±0.05**^e^**	1.09±0.03**^d^**	72.2±1.48**^e^**	22.1±0.46**^e^**
	DBH**_6.0_**	14.8±0.38**^d^**	38.9±0.98**^d^**	2.54±0.05**^d^**	1.24±0.04**^c^**	75.5±1.50**^d^**	24.3±0.49**^d^**
SW	SCt	14.4±0.40**^c^**	44.2±1.24**^c^**	2.77±0.06**^c^**	1.24±0.04**^c^**	78.7±1.64**^c^**	26.2±0.52**^c^**
	DLFJ**_4.0_**	15.4±0.42**^b^**	48.4±1.38**^b^**	2.96±0.06**^b^**	1.35±0.04**^b^**	84.0±1.80**^b^**	28.2±0.56**^b^**
	DBH**_6.0_**	17.8±0.50**^a^**	54.6±1.46**^a^**	3.24±0.07**^a^**	1.62±0.05**^a^**	88.3±1.94**^a^**	31.8±0.64**^a^**
*p*-value		0.011*	0.003**	0.001**	<0.001**	0.030*	0.050*

**^1^** Based on the LSD test, mean values (± standard errors) followed with similar letters in the same column not differed significantly at *p* ≤ 0.05 level of probability. IrrW, irrigation water; Bio-Ss, biostimulators; EC_w_, irrigation water salinity; NCt, normal control; where seeds were soaked and then irrigated using normal water, SCt; saline control, where seeds soaked in normal water and then irrigated with saline water, DLFJ_4.0%_; soaking the seeds in diluted lemon juice at a concentration of 4.0%, DBH_6.0%_; soaking the seeds in diluted bee honey solution at a concentration of 6.0%, TSsg, total soluble sugars; FPro, free proline; AsA, ascorbate; GSH, glutathione; µM, micromole; FW, fresh weight; and DW, dry weight.

*p < 0.05 (statistically significant); **p < 0.01 (highly statistically significant).

### Impacts of DLFJ_4%_ or DBH_6%_ on enzymes and total antioxidant activity in soybean

Irrigating soybean seedlings with saline water significantly increased the activities of several antioxidant enzymes, including superoxide dismutase (SOD), catalase (CAT), ascorbate peroxidase (APX), and glutathione reductase (GR). This saline treatment also markedly enhanced total antioxidant activity (TAA) while reducing the soluble protein (SP) content compared to the NCt. Seed priming in DLFJ_4%_ and DBH_6%_ further elevated the activities of SOD, CAT, APX, and GR, as well as TAA and SP content, thereby helping to mitigate the negative effects of salinity (**Table 5**). Compared to irrigation with normal water (NCt), irrigating seedlings with saline water led to an increase of 31% in SOD activity, 31% in CAT activity, 15% in APX activity, 23% in GR activity, 16% in TAA, and 34% in SP content. Moreover, priming soybean seeds in DLFJ_4%_ and DBH_6%_, and irrigated with normal water, led to an increase of 5% and 17% in SOD activity, 4% and 15% in CAT activity, 5% and 9% in APX activity, 4% and 10% in GR activity, 4% and 8% in TAA, and 7% and 13% in SP content, respectively, compared to the NCt. Furthermore, seed priming in DLFJ_4%_ and DBH_6%_, and irrigated with saline water, resulted in an increase of 7% and 16% in SOD activity, 6% and 17% in CAT activity, 6% and 15% in APX activity, 8% and 17% in GR activity, 6% and 12% in TAA, and 28% and 45% in SP content, respectively, compared to the SCt.

**Table 5:** Response of enzyme and total antioxidant activity in soybean (*Glycine max* L., cv. Giza-111) seedlings grown under irrigation with normal water (NW; EC_w_ = 1.60 dS m^–1^) or saline water (SW; EC_w_ = 8.60 dS m^–1^) to soak the seeds in diluted lemon fruit juice (DLFJ) or diluted bee honey (DBH) at 4.0 or 6.0%, respectively.

Treatments^1^		SOD	CAT	APX	GR	SP content	Antioxidant activity
Irrw	Bio-ss	(u g^–1^ protein)				(mg g^–1^ FW)	(%)
NW	NCt	20.4±0.42**^f^**	16.2±0.33**^f^**	24.8±0.48**^f^**	22.1±0.44**^f^**	155±3.7**^c^**	40.2±0.96**^f^**
	DLFJ**_4.0_**	21.4±0.43**^e^**	16.9±0.35**^e^**	26.0±0.51**^e^**	22.9±0.45**^e^**	166±3.9**^b^**	41.8±1.05**^e^**
	DBH**_6.0_**	23.8±0.46**^d^**	18.6±0.38**^d^**	27.0±0.54**^d^**	24.2±0.47**^d^**	175±4.0**^a^**	43.5±1.12**^d^**
SW	SCt	26.8±0.51**^c^**	21.2±0.40**^c^**	28.6±0.56**^c^**	27.2±0.51**^c^**	102±3.3**^e^**	46.8±1.20**^c^**
	DLFJ**_4.0_**	28.6±0.58**^b^**	22.5±0.43**^b^**	30.2±0.59**^b^**	29.4±0.56**^b^**	131±3.5**^d^**	49.8±1.24**^b^**
	DBH**_6.0_**	31.0±0.64**^a^**	24.8±0.49**^a^**	32.8±0.65**^a^**	31.9±0.64**^a^**	148±3.6**^c^**	52.6±1.30**^a^**
*p*-value		0.005**	0.003**	0.005**	0.001**	0.025*	0.030*

**^1^** Based on the LSD test, mean values (± standard errors) followed with similar letters in the same column not differed significantly at *p* ≤ 0.05 level of probability. IrrW, irrigation water; Bio-Ss, biostimulators; ECw, irrigation water salinity; NCt, normal control; where seeds were soaked and then irrigated using normal water, SCt, saline control; where seeds soaked in normal water and then irrigated with saline water, DLFJ_4.0%_, soaking the seeds in diluted lemon juice at a concentration of 4.0%; DBH_6.0%_, soaking the seeds in diluted bee honey solution at a concentration of 6.0%; SOD, superoxide dismutase; CAT, catalase; APX, ascorbate peroxidase; GR, glutathione reductase; SP, soluble protein; and FW, fresh weight.

*p < 0.05 (statistically significant); **p < 0.01 (highly statistically significant).

### Impacts of DLFJ_4%_ or DBH_6%_ on the nutritional status of soybean

Salinity stress significantly declined nutrient contents, including nitrogen (N), phosphorus (P), magnesium (Mg), iron (Fe), manganese (Mn), zinc (Zn), potassium (K^+^), and calcium (Ca^2+^), as well as K^+^/Na^+^ and Ca^2+^/Na^+^ ratios. In contrast, it significantly increased Na^+^ content compared to the NCt. However, seed priming in DLFJ_4%_ and DBH_6%_ significantly improved the contents of these nutrients while decreasing Na^+^ content, helping to mitigate the adverse effects of salinity (**Tables 6** and **7**). Irrigating seedlings with saline water resulted in a decrease of 33% in N content, 33% in P content, 48% in Mg content, 39% in Fe content, 38% in Mn content, 38% in Zn content, 33% in K^+^ content, 39% in Ca^2+^ content, 84% in K^+^/Na^+^ ratio, and 86% in Ca^2+^/Na^+^ ratio. In contrast, Na^+^ content increased by 320% compared to the NCt. However, seed priming in DLFJ_4%_ and DBH_6%_ and irrigating with normal water led to an increase of 4% and 10% in N content, 4% and 10% in P content, 5% and 11% in Mg content, 7% and 15% in Fe content, 6% and 12% in Mn content, 6% and 14% in Zn content, 4% and 10% in K^+^ content, 4% and 10% in Ca^2+^ content, 5% and 12% in K^+^/Na^+^ ratio, and 5% and 13% in Ca^2+^/Na^+^ ratio, while Na^+^ content slightly decreased by 1% and 2%, respectively, compared to the NCt. Furthermore, seed priming in DLFJ_4%_ and DBH_6%_ and irrigated with saline water led to a significant increase of 31% and 44% in N content, 24% and 48% in P content, 66% and 88% in Mg content, 38% and 63% in Fe content, 35% and 57% in Mn content, 36% and 62% in Zn content, 24% and 46% in K^+^ content, 16% and 58% in Ca^2+^ content, 116% and 410% in K^+^/Na^+^ ratio, and 104% and 457% in Ca^2+^/Na^+^ ratio, while Na^+^ content decreased by 43% and 71%, respectively, compared to the SCt.

**Table 6:** Response of nutritional status of soybean (*Glycine max* L., cv. Giza-111) seedlings grown under irrigation with normal water (NW; EC_w_ = 1.60 dS m^–1^) or saline water (SW; EC_w_ = 8.60 dS m^–1^) to soak the seeds in diluted lemon fruit juice (DLFJ) or diluted bee honey (DBH) at 4.0 or 6.0%, respectively.

Treatments^1^		N	P	Mg	Fe	Mn	Zn
Irrw	Bio-ss	Mg g^–1^ DW			Mg kg^–1^ DW		
NW	NCt	20.2±0.49**^c^**	1.86±0.04**^c^**	5.12±0.13**^c^**	224±5.9**^c^**	152±3.7**^c^**	98.6±2.7**^c^**
	DLFJ**_4.0_**	21.1±0.52**^b^**	1.94±0.05**^b^**	5.38±0.14**^b^**	239±6.2**^b^**	161±3.8**^b^**	104.8±2.9**^b^**
	DBH**_6.0_**	22.2±0.55**^a^**	2.05±0.05**^a^**	5.66±0.16**^a^**	258±6.3**^a^**	170±3.9**^a^**	112.8±2.9**^a^**
SW	SCt	13.6±0.35**^e^**	1.24±0.03**^e^**	2.66±0.07**^e^**	136±3.5**^e^**	94.5±2.6**^e^**	61.2±2.1**^e^**
	DLFJ**_4.0_**	17.8±0.39**^d^**	1.54±0.04**^d^**	4.42±0.10**^d^**	188±4.9**^d^**	128±3.0**^d^**	83.4±2.4**^d^**
	DBH**_6.0_**	19.6±0.48**^c^**	1.83±0.04**^c^**	4.99±0.12**^c^**	221±5.7**^c^**	148±3.8**^c^**	99.0±2.8**^c^**
*p*-value		0.015*	0.030*	0.050*	0.011*	0.005**	0.050*

**^1^** Based on the LSD test, mean values (± standard errors) followed with similar letters in the same column not differed significantly at *p* ≤ 0.05 level of probability. IrrW, irrigation water; Bio-Ss, biostimulators; ECw, irrigation water salinity; NCt, normal control; where seeds were soaked and then irrigated using normal water, SCt, saline control; where seeds soaked in normal water and then irrigated with saline water, DLFJ_4.0%_, soaking the seeds in diluted lemon juice at a concentration of 4.0%; DBH_6.0%_, soaking the seeds in diluted bee honey solution at a concentration of 6.0%; N, nitrogen; P, phosphorus; Mg, magnesium; Fe, iron; Mn, manganese; Zn, zinc; and DW, dry weight.

*p < 0.05 (statistically significant); **p < 0.01 (highly statistically significant).

**Table 7:** Response of nutritional status of soybean (*Glycine max* L., cv. Giza-111) seedlings grown under irrigation with normal water (NW; EC_w_ = 1.60 dS m^–1^) or saline water (SW; EC_w_ = 8.60 dS m^–1^) to soak the seeds in diluted lemon fruit juice (DLFJ) or diluted bee honey (DBH) at 4.0 or 6.0%, respectively.

Treatments^1^		K^+^ content	Ca^2+^ content	Na^+^ content	K^+^/Na^+^ ratio	Ca^2+^/Na^+^ ratio
Irrw	Bio-ss	Mg g^–1^ DW	Mg kg^–1^ DW			
NW	NCt	21.4±0.52**^c^**	5.60±0.14**^c^**	3.52±0.10**^d^**	6.08±0.17**^c^**	1.59±0.05**^c^**
	DLFJ**_4.0_**	22.3±0.55**^b^**	5.84±0.15**^b^**	3.50±0.08**^d^**	6.37±0.19**^b^**	1.67±0.06**^b^**
	DBH**_6.0_**	23.5±0.54**^a^**	6.15±0.17**^a^**	3.44±0.09**^d^**	6.83±0.19**^a^**	1.79±0.08**^a^**
SW	SCt	14.4±0.31**^e^**	3.44±0.10**^e^**	14.8±0.46**^a^**	0.97±0.06**^f^**	0.23±0.01**^f^**
	DLFJ**_4.0_**	17.8±0.49**^d^**	3.98±0.12**^d^**	8.48±0.30**^b^**	2.10±0.10**^e^**	0.47±0.02**^e^**
	DBH**_6.0_**	21.0±0.54**^c^**	5.42±0.13**^c^**	4.24±0.18**^c^**	4.95±0.13**^d^**	1.28±0.04**^d^**
*p*-value		0.005**	0.035*	0.003**	0.001**	0.030*

**^1^** Based on the LSD test, mean values (± standard errors) followed with similar letters in the same column not differed significantly at *p* ≤ 0.05 level of probability. IrrW, irrigation water; Bio-Ss, biostimulators; ECw, irrigation water salinity; NCt, normal control; where seeds were soaked and then irrigated using normal water, SCt, saline control; where seeds soaked in normal water and then irrigated with saline water, DLFJ_4.0%_, soaking the seeds in diluted lemon juice at a concentration of 4.0%; DBH_6.0%_, soaking the seeds in diluted bee honey solution at a concentration of 6.0%; K^+^, potassium ion; Ca^2+^, calcium ion; Na^+^, sodium ion; and DW, dry weight.

*p < 0.05 (statistically significant); **p < 0.01 (highly statistically significant).

### Impacts of DLFJ+DBH on phytohormone contents of soybean

**Figure 4:**
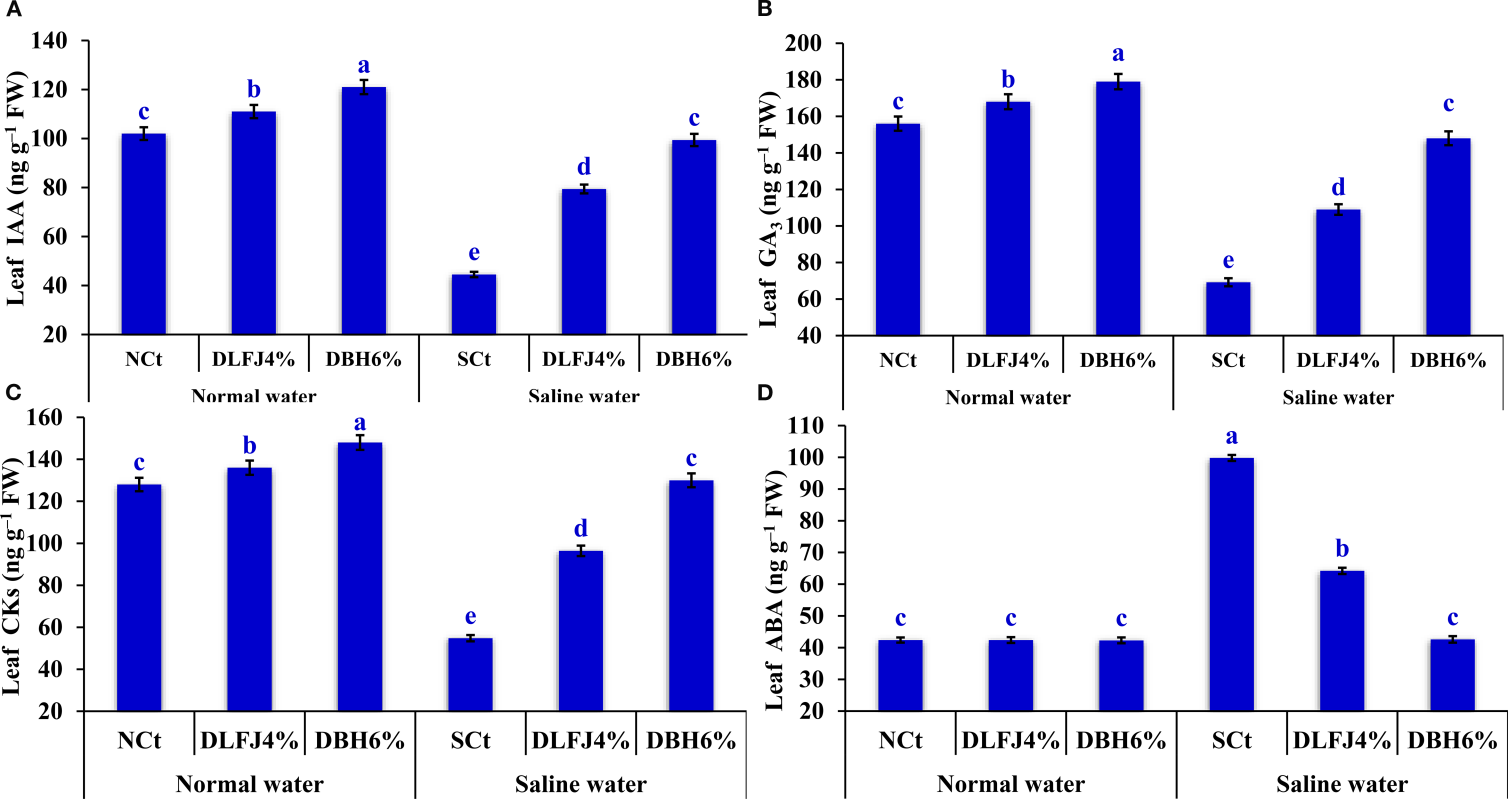
Response of phytohormone contents; IAA **(A)**, GA_3_ **(B)**, CKs **(C)**, and ABA **(D)** of soybean seedlings (*Glycine max* L., cv. Giza-111) grown under irrigation with normal water (EC_w_= 1.60 dS m^–1^) or saline water (EC_w_= 8.60 dS m^–1^) to soaking the seeds in DLFJ_4%_ or DBH_6%_. Based on the two-way analysis of variance conducted across all six treatment combinations (i.e., irrigation water saline × foliar biostimulant) and the LSD test, bars (mean ± standard error; n = 3 for each) labeled with similar letters in the same column did not differ significantly at a *p* ≤ 0.05 level of probability. NCt, normal control; where seeds were soaked and then irrigated using normal water, SCt, saline control; where seeds soaked in normal water and then irrigated with saline water, DLFJ_4.0%_, soaking the seeds in diluted lemon juice at a concentration of 4.0%; DBH_6.0%_, soaking the seeds in diluted bee honey solution at a concentration of 6.0%; GP, the germination percentage measured when the seed radicle reaches about 2 mm in length; IAA, indole-3-acetic acid; GA_3_, gibberellic acid; CKs, cytokinins; ABA, abscisic acid; FW, fresh weight; and ECw, irrigation water salinity.

Irrigating soybean seedlings with saline water negatively impacted the levels of several phytohormones, including indole-3-acetic acid (IAA), gibberellic acid (GA_3_), cytokinins (CKs), and abscisic acid (ABA). However, seed priming in DLFJ_4%_ and DBH_6%_ significantly restored the balance of these phytohormones, which were associated with seedling growth and nutrient uptake, and mitigated the adverse effects of salinity (**Figure 4**). Irrigating seedlings with saline water caused a decline of 56% in IAA content, 56% in GA_3_ content, and 57% in CKs content, while ABA increased by 135% compared to the NCt. However, seed priming in DLFJ_4%_ and DBH_6%_ and irrigated with normal water caused an increase of 9% and 19% in IAA content, 8% and 15% in GA_3_ content, and 6% and 16% in CKs, respectively, compared to the NCt. There were no changes observed in ABA content. Furthermore, seed priming in DLFJ_4%_ and DBH_6%_ and irrigating with saline water caused an increase of 78% and 123% in IAA content, 58% and 114% in GA_3_ content, and 76% and 137% in CKs content, respectively. In contrast, ABA content was reduced by 36% and 57%, respectively, compared to the SCt.

## Discussion

This study investigated the improvement of soybean seedling performance under irrigation with saline water (EC_w_ = 8.60 dS m^–1^) through seed priming using promising natural biostimulants (NBSs), specifically DLFJ_4%_ and DBH_6%_. These NBSs are rich in bioactive compounds that effectively promote seed priming for cultivation in saline-affected regions. This approach has successfully supported the growth of *Glycine max* seedlings under salt stress by improving their tolerance to high salt concentrations. Nazari et al. (2023) stated that salinity stress significantly decreases the percentage of seed germination (GP). High salt levels severely decrease plant fresh and dry weights and root activity. In addition, severe salt stress can significantly reduce growth strength and hinder GP and early seedling development across various species (Mogazy and Hanafy, 2022). The hindrance of seed GP under salt stress is attributed to reduced water availability for seed embryo growth. Salinity also inhibits starch movement to the seed endosperm and limits the transport of soluble sugars to the seed embryonic axis (Chakrobortty et al., 2022; Haider et al., 2023). However, seed priming in NBSs (e.g., DLFJ_4%_ and DBH_6%_ in this study) enhance seed GP and reduce the negative effects of salt. This improvement is due to the beneficial bioactive components found in these NBSs. The bioactive components of the NBSs help plants adapt to salinity by activating metabolic pathways and salt tolerance genes, increasing root activity, and enhancing seedling growth (Tahjib-Ul-Arif et al., 2021; Rady et al., 2023a).

Salt stress adversely affects leaf integrity traits, measured as RWC and MSI. This may occur because plants subjected to salt stress undergo water loss resulting from ionic imbalance and osmotic stress (Alsamadany et al., 2023). However, seed priming in NBSs (e.g., DLFJ_4%_ and DBH_6%_ in this study) markedly improves RWC and MSI. In this respect, the DBH_6%_ treatment showing greater effectiveness than the DLFJ_4%_ treatment. Seed priming in DLFJ_4%_ and DBH_6%_ help maintain the structural integrity of cell membranes under salt stress. This preservation explains the observed improvements in RWC and MSI (Belal et al., 2023; Rady et al., 2023a).

Alsamadany et al. (2023) reported that salinity stress significantly reduces the photosynthetic apparatus, as indicated by the contents of TChls and TCars, as well as *F_v_/F_m_*, PI, *Pn*, *A*, *gs*, and *E*. However, NBSs (e.g., DLFJ_4%_ and DBH_6%_ in this study) significantly improve these parameters by mitigating the harmful impacts of salinity. Alsamadany et al. (2023) stated that salinity stress decreases the biosynthesis of pigments by inhibiting the activity of several enzymes, including 5-aminolevulinic acid dehydratase, porphobilinogen deaminase, coproporphyrinogen III oxidase, porphyrinogen IX oxidase, Mg-chelatase, and protochlorophyllide oxidoreductase. The salinity-induced reduction of pigments could be attributed to increased production of ethylene caused by higher levels of Na^+^ and Cl^–^ and overproduction of ROS. Furthermore, nutritional limitation and oxidative damage occur under salt stress conditions (Azzam et al., 2022; Rady et al., 2023b). This study confirms that priming soybean seeds with DLFJ_4%_ and DBH_6%_ reduces the negative effects of salinity and improves the performance of the photosynthesis apparatus. The NBSs enrich the seeds and then the plants with osmolytes and essential nutrients, which enhance the antioxidant system, reduce ROS levels and lipid peroxidation, preserve chlorophyll integrity, and mitigate salt stress (Alghamdi et al., 2022; Rady et al., 2023a).

Under salt stress conditions, the levels of ROS, such as O_2_^•–^ and H_2_O_2_, increase. Additionally, this stress leads to increased lipid peroxidation, as indicated by levels of MDA and EL. This increase in ROS levels due to salt stress limits plant growth by degrading chlorophyll, lipids, proteins, and DNA, leading to cell damage and death (Abou-Sreea et al., 2021; Chauhan et al., 2023). MDA and EL serve as notable measures of oxidative stress caused by membrane damage, which further hampers cellular function and has detrimental effects on plant growth and productivity. Lipid peroxidation caused by ROS is due to their negative effects on the polyunsaturated fatty acids in cell membranes, leading to alterations in the structure and function of the membrane (Sayed et al., 2024). Seed priming in NBSs (e.g., DLFJ_4%_ and DBH_6%_ in this study) helps mitigate the negative impacts of salinity by lowering the levels of ROS (e.g., O_2_> ^•–^ and H_2_O_2_) and stress damage, as indicated by MDA and EL. The lower levels of ROS and stress damage are attributed to improved enzymatic and non-enzymatic antioxidant systems along with osmoregulation mechanisms, which enhance membrane stability and preserve cell membrane integrity (Alharby et al., 2021; Hasanuzzaman et al., 2022; Rady et al., 2023a).

Zaid and Wani (2019) demonstrated that salinity stress leads to an increase in the accumulation of total soluble sugars (TSsg), free proline (FPro), AsA, and GSH in plants, alongside the redox states of AsA and GSH in plants. The accumulation of TSsg and FPro can be attributed to decreased water absorption under salt stress. GSH plays a crucial role in protecting cell membranes from oxidative stress caused by ROS. It does this by preserving zeaxanthins and tocopherols in their reduced forms, which helps safeguard the membranes. Additionally, GSH protects proteins from denaturation by inhibiting the oxidation of protein thiol groups under salt stress conditions (Zaid and Wani, 2019). The loss of membrane integrity under salt stress appears to create conditions similar to drought, resulting in increased levels of proline. These findings suggest that salt-stressed plants experience more damage, requiring higher levels of TSsg and FPro to maintain cellular osmotic balance. Additionally, exposure to salinity triggers the expression of genes related to drought (Tanveer and Ahmed, 2020; Balasubramaniam et al., 2023). Treatment with NBSs (e.g., DLFJ_4%_ and DBH_6%_ in this study) significantly improves these parameters under stress, helping to maintain osmotic pressure, enhance water absorption, protect cells from damage, stabilize subcellular structures and proteins, eliminate ROS, and increase salt stress tolerance (Desoky et al., 2021; Rady et al., 2023a).

Irin and Hasanuzzaman (2024) concluded that salinity stress leads to the overproduction of ROS, which can severely impact plant growth as well as physio-biochemical and metabolic processes. To alleviate the detrimental impacts of salinity stress, plants activate their antioxidant defense systems, which includes both non-enzymatic (FPro, AsA, and GSH) and enzymatic antioxidants (SOD, CAT, APX, and GR) that neutralize ROS (Canavar et al., 2023). SOD acts as the primary defense against O_2_^•–^ by transforming it into H_2_O_2_. Peroxidases then convert H_2_O_2_ into O_2_ and H_2_O. In most living organisms, CAT is primarily located in cell peroxisomes, where it has a higher affinity for H_2_O_2_ and effectively breaks it down. In the Halliwell-Asada cycle, APX is considered the most crucial enzyme in lowering ROS levels (Irin and Hasanuzzaman, 2024). Additionally, GR and DHAR are recognized as essential enzymes in the Halliwell-Asada cycle for regeneration. They facilitate the regeneration of AsA from DHA by utilizing GSH as a reducing agent (Rady et al., 2021). In salt-stressed plants, the activity of enzymatic antioxidants was found to increase. However, seed priming in MBSs (e.g., DLFJ_4%_ and DBH_6%_ in this study) further increased antioxidant levels and enzyme activity in salt-stressed plants. This supplemental approach appears to enhance antioxidant defense and osmoregulation mechanisms in response to salinity stress by altering cell osmosis, stabilizing membrane structures, and minimizing oxidative stress damage (Franzoni et al., 2022; Canavar et al., 2023; Tarfayah et al., 2023).

Salt stress adversely affected the plant nutritional status, leading to a significant increase in Na^+^ content while reducing levels of essential nutrients (N, P, Mg, Fe, Mn, Zn, K^+^, and Ca^2+^). It influences the uptake of minerals by changing the mineral content in the soil (Dichala et al., 2022). It can also decrease plant mineral content by disrupting their uptake, primarily due to its impact on the permeability of plasma membranes. Furthermore, it negatively impacts stomatal conductivity, respiration, and photosynthesis efficiency (Desoky et al., 2021). Increased concentrations of Na^+^ and Cl^–^ during salt stress facilitate ion transport into plant tissues, resulting in oxidative damage, osmotic imbalance, and molecular damage. This leads to harmful effects on the metabolic processes of both cell membranes and cytosol (Haider et al., 2023). However, the introduction of DLFJ_4%_ and DBH_6%_ supplements in this study was found to enhance stability of cell membrane. This finding may be attributed to the increased K^+^/Na^+^ ratio and Ca^2+^/Na^+^ ratios and osmoregulatory compound contents (Jamali et al., 2015). These NBSs enhance nutrient uptake and reduce Na^+^ absorption, limiting its movement to the above-ground parts of the plants. The increase in nutrient contents (N, P, Mg, Fe, Mn, Zn, K^+^, and Ca^2+^) and the decrease in Na^+^ content confirm the rise in both K^+^/Na^+^ and Ca^2+^/Na^+^ ratios (Semida et al., 2019; Irin and Hasanuzzaman, 2024).

Salt-stressed plants display an increase in ABA levels, while IAA, GA_3_, and CKs decrease significantly. However, seed priming in NBSs (e.g., DLFJ_4%_ and DBH_6%_ in this study) increases IAA, GA_3_, and CKs levels, while ABA level decreases (Belal et al., 2023). The DBH_6%_ treatment is significantly superior to the DLFJ_4%_ treatment in this study. Physio-biochemical processes are upregulated in plants by phytohormones even under stress conditions (Balasubramaniam et al., 2023). The improvement in phytohormone content in calendula leaves by applying 5% BHS treatment may be due to the increased content of nutrients necessary for protoplasm and phytohormone formation (Semida et al., 2019; Siddiki et al., 2020). GAs upregulate IAA-related genes and downregulate other ABA-related genes, while CKs possess antagonistic actions against ABA (Sytar et al., 2019; Semida and Rady, 2014). Moreover, DELLA protein degradation, catalyzed by GAs, is harmonized by various signals, including other phytohormones (Rady et al., 2023a). This reveals that GAs interfere with and upregulate other phytohormones to overcome stress. Hormonal balance has been achieved in other BHS-treated plants, both under normal and stressful growing conditions (Rady et al., 2023b; Sayed et al., 2024).

## Conclusions and future prospects

Natural biostimulants (NBSs) play an important role in sustainable agriculture, particularly in regions affected by salinity. Rich in bioactive compounds, specific NBSs such as DLFJ4% and DBH6% offer a promising method for enhancing seed priming in these challenging environments. Seed priming with DLFJ_4%_ and DBH_6%_ has proven effective in supporting the growth of *Glycine max* seedlings under salt stress by improving their tolerance to high salinity levels. Data demonstrated that DBH_6%_ exhibited superior efficacy compared to DLFJ_4%_. The findings of this study show that seed priming in DBH_6%_ was the most effective treatment for reducing the damage caused by salinity to the morpho-physiological characteristics of *Glycine max* plants. This treatment improved the K^+^/Na^+^ homeostasis, non-enzymatic and enzymatic antioxidant activities, osmoprotectants, and hormonal levels. It also decreased the Na^+^ and Cl^−^ level, abscisic acid content, and oxidative stress biomarkers-related membrane damage. Thus, DBH_6%_ can serve as a cost-effective biostimulant or a source of micro-element nutrients for plants in both normal and stressed conditions, offering a more affordable alternative to costly synthetic chemicals, such as fertilizers, phytohormones, or antioxidants. Further research is necessary to understand the precise mechanisms of DBH as a natural biostimulant in regulating various cell signaling pathways and physiological responses under environmental stressors.

Recent advancements in sustainable agriculture have shown that solutions made from bee honey and lemon juice can effectively enhance crop yield and improve stress tolerance. Improper concentration and application of these biostimulants in farming practices may lead to unsatisfactory results. Different crop plants may react differently to solutions of bee honey and lemon juice, which can lead to potential phytotoxicity that depends on the dosage. Besides the numerous advantages, it’s important to consider the limitations of these biostimulants before incorporating them into agricultural recommendations. However, utilizing these biostimulants can significantly enhance food safety, with positive implications for human health and food security. Although this study yielded promising results, additional research under field conditions and over extended growth periods is needed to confirm these findings. Furthermore, more physio-biochemical and molecular studies are essential to fully understand how the bioactive components in these biostimulants interact with plant cells and their specific mechanisms.

## Data Availability

The original contributions presented in the study are included in the article/**Supplementary Material**. Further inquiries can be directed to the corresponding authors.
