# Supplementary material for: Role of seed priming using natural biostimulants in reducing salt stress effects by reshaping physio-biochemical and antioxidant defense systems in glycine max seedlings
Source: Front Plant Sci. 2025 Oct 13;16:1630537. doi: 10.3389/fpls.2025.1630537 (PMC12554584; doi:10.3389/fpls.2025.1630537)
Supplement: Supplementary file 1 [file Table1.docx]

**Table S1.** A preliminary trial to identify the optimal priming duration for soybean (*Glycine max* L., cv. Giza-111) seeds to be used in the main trial

| **Treatment** | **GP** | **Seedling FW** | **Seedling DW** | **TChls content** |
| --- | --- | --- | --- | --- |
| Soaking duration | % | g | | mg g^‒1^ FW |
| 2h | 73.2±2.34c | 2.14±0.14d | 0.21±0.01d | 1.68±0.06c |
| 4h | 77.6±2.90b | 2.56±0.17b | 0.28±0.02b | 1.84±0.11b |
| 6h | 78.2±3.00b | 2.58±0.19b | 0.29±0.02b | 1.90±0.09b |
| 8h | 82.8±3.22a | 2.98±0.21a | 0.36±0.02a | 2.20±0.11a |
| 10h | 82.4±3.20a | 2.94±0.20a | 0.34±0.02a | 2.15±0.11a |
| 12h | 72.6±2.91c | 2.33±0.16c | 0.25±0.02c | 1.76±0.06c |
| *p*-value | 0.018* | 0.001** | <0.001** | 0.005** |

Based on the LSD test, mean values (± standard errors) followed with similar letters in the same column not differed significantly at *p* ≤ 0.05 level of probability. GP; germination percentage, FW; fresh weight, DW; dry weight, and TChls; total chlorophylls.

**Table S2.** A preliminary trial to identify the optimal level of diluted lemon fruit juice (DLFJ) for soaking soybean (*Glycine max* L., cv. Giza-111) seeds to be used in the main trial

| **Treatment** | **GP** | **Seedling FW** | **Seedling DW** | **TChls content** |
| --- | --- | --- | --- | --- |
| DLFJ levels | % | g | | mg g^‒1^ FW |
| 2% | 72.9±2.32**^b^** | 2.10±0.13**^b^** | 0.20±0.01**^b^** | 1.72±0.06**^b^** |
| 4% | 86.4±3.50**^a^** | 3.05±0.22**^a^** | 0.38±0.02**^a^** | 2.10±0.10**^a^** |
| 6% | 85.0±3.44**^a^** | 3.02±0.21**^a^** | 0.37±0.02**^a^** | 2.08±0.10**^a^** |
| 8% | 70.8±2.22**^b^** | 1.98±0.12**^b^** | 0.20±0.01**^b^** | 1.60±0.05**^c^** |
| *p*-value | 0.021* | <0.001** | <0.001** | 0.001** |

Based on the LSD test, mean values (± standard errors) followed with similar letters in the same column not differed significantly at *p* ≤ 0.05 level of probability. GP; germination percentage, FW; fresh weight, DW; dry weight, and TChls; total chlorophylls.

**Table S3.** A preliminary trial to identify the optimal level of diluted bee honey (DBH) for soaking soybean (*Glycine max* L., cv. Giza-111) seeds to be used in the main trial

| **Treatment** | **GP** | **Seedling FW** | **Seedling DW** | **TChls content** |
| --- | --- | --- | --- | --- |
| DBH levels | % | g | | mg g^‒1^ FW |
| 3% | 62.2±2.14**^c^** | 2.10±0.12**^c^** | 0.20±0.01**^c^** | 1.68±0.06**^c^** |
| 6% | 89.4±3.74**^a^** | 3.48±0.31**^a^** | 0.44±0.02**^a^** | 2.66±0.12**^a^** |
| 9% | 89.6±3.80**^a^** | 3.46±0.29**^a^** | 0.44±0.02**^a^** | 2.60±0.11**^a^** |
| 12% | 80.5±3.00**^b^** | 2.80±0.18**^b^** | 0.32±0.02**^b^** | 2.24±0.09**^b^** |
| *p*-value | 0.010* | <0.001** | <0.001** | <0.001** |

Based on the LSD test, mean values (± standard errors) followed with similar letters in the same column not differed significantly at *p* ≤ 0.05 level of probability. GP; germination percentage, FW; fresh weight, DW; dry weight, and TChls; total chlorophylls.
